# Therapeutic effects of focused ultrasound-mediated blood-brain barrier opening in a mouse model of Alzheimer’s disease

**DOI:** 10.1186/2050-5736-3-S1-O16

**Published:** 2015-06-30

**Authors:** Alison Burgess, Sonam Dubey, Tam Nhan, Isabelle Aubert, Kullervo Hynynen

**Affiliations:** 1 Sunnybrook Research Institute, Toronto, Ontario, Canada

## Background/introduction

Focused ultrasound (FUS)-mediated opening of the blood-brain barrier (BBB) has been used to promote drug delivery to the brain. However, in a mouse model of Alzheimer’s disease, FUS has proven to be effective for reducing pathology and improving cognition, even in the absence of exogenous drug application. We have explored single and repeated FUS treatments in mice at both early and late stages of plaque pathology using a transgenic (Tg) mouse model of Alzheimer’s disease.

## Methods

Mice received single or weekly FUS treatments using a 1.68 MHz transducer, (10 ms pulses, 1Hz pulse repetition frequency, 120 second duration). Definity microbubble contrast agent was delivered at the onset of sonication. Effective BBB opening was confirmed using contrast enhanced MR images. Following FUS treatments, mice were evaluated using cognitive tests and histology.

## Results and conclusions

In the Y maze with novel arm, untreated Tg mice were found to spend significantly less time in the novel arm compared to their non-Tg littermates.

Following FUS treatment, Tg mice performed as well as their non-Tg counterparts. These results indicate that repeated FUS treatments are safe and may improve cognitive behavior, even in a model of late-stage AD. To support these findings, mice treated with FUS also had reduced plaque burden in the brain and increased plasticity in the hippocampus, both of which have been correlated to improved learning and memory. Together this data demonstrates that FUS-mediated BBB opening can improve cognition related even in the absence of exogenous drug delivery and suggests that FUS should be considered part of the overall therapeutic strategy for treatment of Alzheimer’s disease.

